# Shear Bond Strength of Orthodontic Brackets and Disinclusion Buttons: Effect of Water and Saliva Contamination

**DOI:** 10.1155/2013/180137

**Published:** 2013-05-15

**Authors:** Maria Francesca Sfondrini, Danilo Fraticelli, Paola Gandini, Andrea Scribante

**Affiliations:** Università degli Studi di Pavia, Dipartimento di Scienze Clinico-Chirurgiche, Diagnostiche e Pediatriche, Sezione di Odontoiatria, Reparto di Ortognatodonzia, Piazzale Golgi 2, 27100 Pavia, Italy

## Abstract

*Purpose*. The aim of this study was to assess the effect of water and saliva contamination on the shear bond strength and failure site of orthodontic brackets and lingual buttons. *Materials and Methods*. 120 bovine permanent mandibular incisors were randomly divided into 6 groups of 20 specimens each. Both orthodontic brackets and disinclusion buttons were tested under three different enamel surface conditions: (a) dry, (b) water contamination, and (c) saliva contamination. Brackets and buttons were bonded to the teeth and subsequently tested using a Instron universal testing machine. Shear bond strength values and adhesive failure rate were recorded. Statistical analysis was performed using ANOVA and Tukey tests (strength values) and Chi squared test (ARI Scores). *Results*. Noncontaminated enamel surfaces showed the highest bond strengths for both brackets and buttons. Under water and saliva contamination orthodontic brackets groups showed significantly lower shear strengths than disinclusion buttons groups. Significant differences in debond locations were found among the groups under the various enamel surface conditions. *Conclusions*. Water and saliva contamination of enamel during the bonding procedure lowers bond strength values, more with orthodontic brackets than with disinclusion buttons.

## 1. Introduction

The procedure of bonding orthodontic brackets to enamel requires completely dry and isolated fields to obtain clinically acceptable bond strengths, because of hydrophobic properties of bonding materials [1]. Many clinical conditions do not permit ideal isolation and moisture contamination is considered the most common reason for bond failure [2, 3]. During many orthodontic procedures (surgical exposure of impacted teeth, rotation movements, and spaces closure) an option is to bond disinclusion buttons to the tooth [4, 5]. The presence of water, saliva, and blood makes it difficult to place an appliance in an isolated field. Therefore, buttons often have to be rebonded which is an unpleasant procedure both for patients and clinicians [1]. 

Previous studies that evaluated the effect of water and saliva contamination on the bond strengths of brackets bonded with light-cured composites showed a significant reduction in bond strength values [1, 6–9]. In literature there are no published studies that evaluated shear bond strength of disinclusion buttons bonded onto water- and saliva-contaminated enamel.

Accordingly, the aim of the present investigation was to measure and compare shear bond strength and adhesive remnant index (ARI) score of a conventional orthodontic bracket and a disinclusion button bonded onto dry, water- and saliva-contaminated enamel. The null hypothesis of the study was that there is no significant difference in shear bond strength values and debond locations among the various groups.

## 2. Material and Methods

One hundred and twenty freshly permanent extracted bovine mandibular incisors were collected from a local slaughter house and stored in a solution of 0.1% (wt/vol) thymol. The criteria for tooth selection included intact buccal enamel with no cracks caused by extraction and no caries. The teeth were cleansed of soft tissue and embedded in cold-curing, fast-setting acrylic (Leocryl, Leone, Sesto Fiorentino, Italy). Metal rings (15-mm diameter) were filled with the acrylic resin and allowed to cure, thus encasing each specimen while allowing the buccal surface of enamel to be exposed. Each tooth was oriented so that its labial surface was parallel to the shearing force. Teeth were randomly divided in six groups of 20 specimens.

Orthodontic stainless steel maxillary central incisor brackets (Leone, Sesto Fiorentino, Italy) and stainless steel orthodontic buttons (Leone, Sesto Fiorentino, Italy) were used. Both brackets and buttons were tested under 3 different enamel surface conditions: (1) dry, (2) water contamination, (3) saliva contamination.

The labial surface of each incisor was cleaned for 10 seconds with a mixture of water and fluoride-free pumice in a rubber polishing cup with a low-speed handpiece. The enamel surface was rinsed with water to remove pumice or debris and then dried with an oil-free air stream. 

Before bonding, 4 scanning electron microscope photographs were taken using a scanning electron microscope (JSM-6480LV, JEOL Ltd, Tokyo, Japan) to observe differences between orthodontic bracket and disinclusion button bases. For each appliance, microphotographs were taken in the most prominent and in the deepest parts of the bracket base (magnification 2500x) [10].

Bonding procedures are described in Table 1. Teeth were etched with 37% phosphoric acid gel (3M Unitek, Monrovia, CA, USA) for 30 seconds, followed by thorough washing and drying. A thin layer of primer (Ortho Solo; Ormco, Glendora, California) was applied on the etched enamel, and then the brackets were bonded with a resin (Transbond XT, 3M Unitek, Monrovia, CA, USA) near the center of the facial surface of the teeth with sufficient pressure to express excess adhesive, which was removed from the margins of the bracket base with a scaler before polymerization. To achieve reproducible conditions, the teeth treated under conditions 2 and 3 were contaminated with water or saliva from a female donor; the moisture was applied with a brush onto the labial surfaces until they were totally contaminated.

Brackets were then light-cured with a visible light-curing unit (Ortholux XT, 3M Unitek, Monrovia, CA, USA) for 10 seconds on the mesial side of the bracket and for 10 seconds on the distal side (total cure time 20 seconds). After bonding, all samples were stored in distilled water at room temperature for 24 hours and then tested in a shear mode on a universal testing machine (Model 4301, Instron, Canton, MA, USA). Specimens were secured in the lower jaw of the machine so that the bonded bracket base was parallel to the shear force direction.

Specimens were stressed in an occlusogingival direction at a crosshead speed of 1 mm per minute, as in previous studies [11–13]. The maximum load necessary to debond or initiate bracket fracture was recorded in newtons and then converted into MPa as a ratio of newtons to surface area of the bracket. After bond failure, the bracket bases and the enamel surfaces were examined under an optical microscope (Stereomicroscope SR, Zeiss, Oberkochen, Germany) at 10x magnification. The adhesive remnant index (ARI) was used to assess the amount of adhesive left on the enamel surface [14]. 

This scale ranges from 0 to 3. A score of 0 indicates no adhesive remaining on the tooth in the bonding area; 1 indicates less than half of the adhesive remaining on the tooth; 2 indicates more than half of the adhesive remaining on the tooth; 3 indicates all adhesive remaining on the tooth. The ARI scores were used as a more complex method of defining bond failure site among the enamel, the adhesive, and the bracket base.

Statistical analysis was performed with Stata 9.0 software (Stata, College Station, TX, USA). Descriptive statistics, including the mean, standard deviation, median, minimum and maximum values were calculated for all groups. 

An analysis of variance (ANOVA) test was applied to determine whether significant differences in debond values existed among the groups. The Tukey test was used as *post hoc*. The chi-square test was performed to determine significant differences in the ARI scores among the different groups. Significance for all statistical tests was predetermined at *P* < 0.05.

## 3. Results

**Figure 1:**
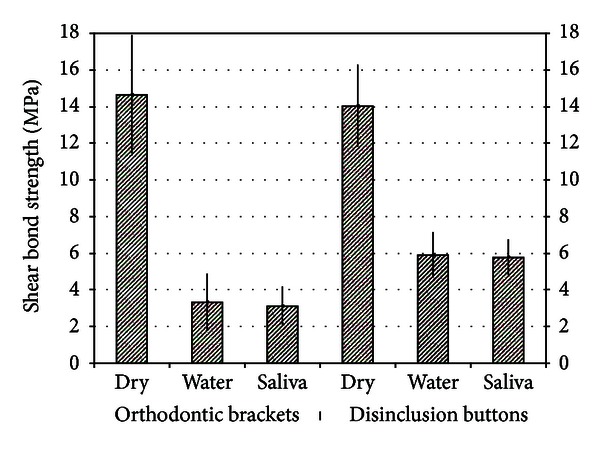
Mean shear bond strengths (MPa) of the two appliances under the three different testing conditions.

Descriptive statistics for the shear bond strength (MPa) of the different brackets are illustrated in Table 2 and Figure 1. The analysis of variance showed the presence of significant differences among the various groups (*P* < 0.05). *Post hoc* test underlined that groups 1 and 4 (brackets and buttons onto dry enamel) had the highest shear bond strength (*P* < 0.001) and exhibited no significant difference between them (*P* > 0.05). Groups 5 and 6 (disinclusion button onto contaminated enamel) had lower bond strengths (*P* < 0.001) and showed no significant difference between them (*P* > 0.05). Groups 2 and 3 (orthodontic bracket onto contaminated enamel) presented the lowest bond strengths and no significant difference among them (*P* > 0.05).

The results of ARI Scores are illustrated in Table 3. The chi-square test reported a higher frequency of ARI score of “2” for uncontaminated enamel groups (1 and 4) (*P* < 0.05) that showed no significant difference between them (*P* > 0.05). Groups 2 and 3 presented higher frequency of ARI score of “0” and no significant difference was found between them (*P* > 0.05). Groups 2 and 3 exhibited higher frequency of ARI score of “1” and no significant difference was found between them (*P* > 0.05).

## 4. Discussion

The null hypothesis of the study has been rejected. In the present investigation conventional brackets and disinclusion buttons bonded onto dry enamel had significantly higher shear bond strength values than other groups and exhibited no significant differences between them. 

**Figure 2:**
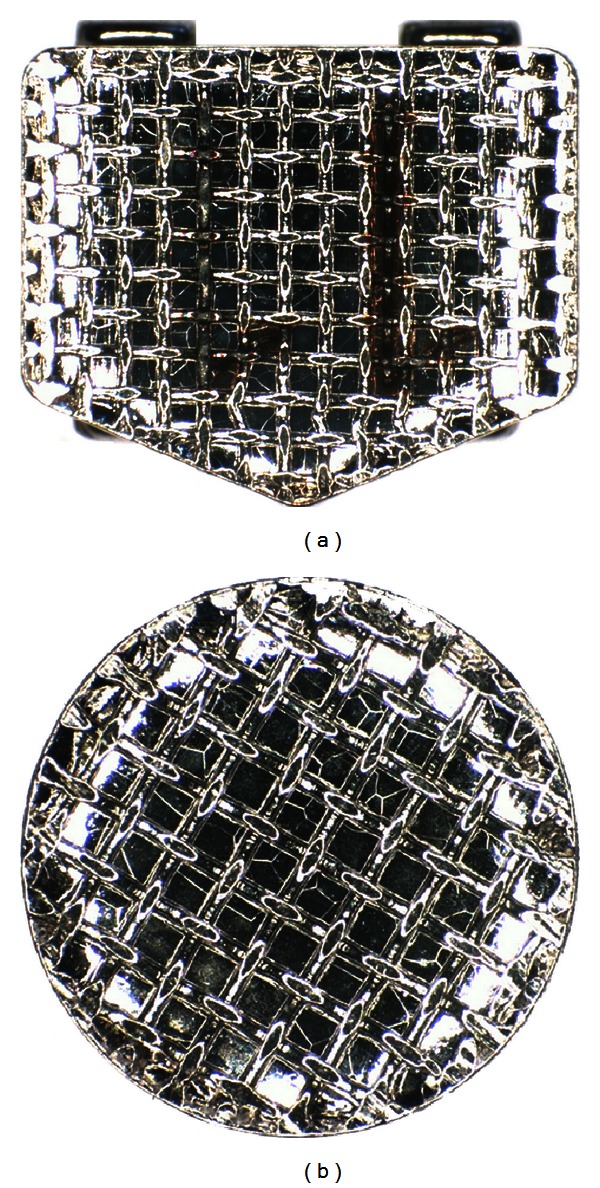
Different bracket (a) and button (b) bases.

**Figure 3:**
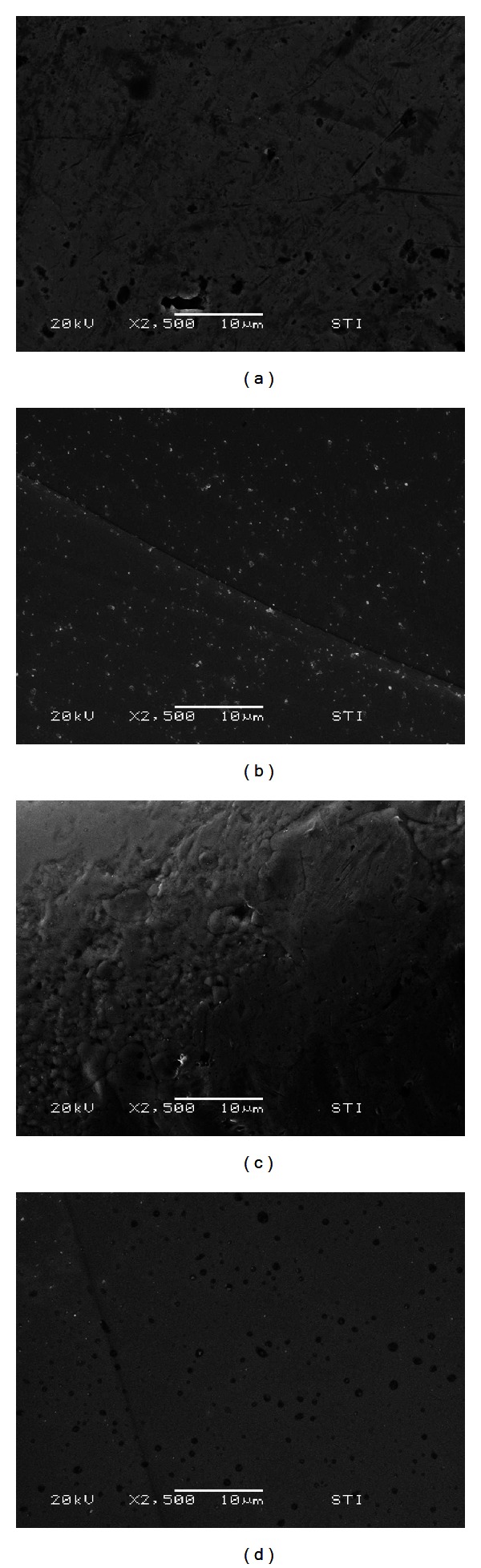
Scanning electron microscopy evaluation (2500x) of the different bases. (a) Orthodontic bracket, most prominent part of the base; (b) bracket, deepest part of the base; (c) disinclusion button, most prominent part of the base; (d) disinclusion button, deepest part of the base.

The similar strength values of the two appliances in dry state is probably due to the similar mesh pad design of the devices tested (Figure 2). Previous investigations [10, 15] showed the relationship between the base of orthodontic brackets and the retention capability. In fact the morphology of the base design may improve the penetration of the adhesive material [16]. Moreover, when evaluating scanning electron microphotographs of the different bases (Figure 3), the orthodontic brackets (Figures 3(a) and 3(b)) showed a similar surface pattern as the disinclusion buttons (Figures 3(c) and 3(d)) both in the most prominent parts than in the recesses of the surface.

Under water- and saliva-contaminated conditions both orthodontic brackets and disinclusion buttons showed significantly lower shear bond strength values. In fact the properties of an adhesive resin can be diminished by various intraoral factors that include high humidity in the oral cavity [6, 17], aging of the tooth [18], dental caries [19] and saliva or blood contamination of the adhesive areas [3, 13, 20]. 

In the present investigation water and saliva contamination of enamel has been investigated. In fact, enamel can be contaminated with water during bonding procedure after conditioning followed by insufficient drying. On the other hand saliva is always present in the patient's oral environment, and it contains antioxidants, immunoglobulins, antibacterial enzymes, growth factors, and mucous secretion to protect epithelial cells from mechanical and chemical challenges [21].

When the etched enamel becomes wet, most of the porosities become plugged, and the penetration of the resin is impaired, which results in resin tags of insufficient number and length [22]. Contamination with water, saliva, and blood has been shown to adversely affect the bond, as deposits an organic adhesive coating within the first few seconds of exposure that is resistant to washing [13, 20, 23].

As blood [1, 4, 9, 13, 20], water [3, 6, 24], and saliva [3, 9, 24] contamination on shear bond strength of orthodontic brackets has been extensively tested, no studies are present about water and saliva contamination of disinclusion buttons. In the present investigation for both disinclusion buttons and orthodontic brackets, no significant differences were found between water- and saliva-contaminated groups. Moreover when tested onto contaminated enamel, disinclusion buttons showed significantly higher shear strengths than conventional brackets. These results agree with those previously reported in another investigations using conventional brackets and disinclusion buttons bonded with blood-moistened enamel surfaces [5].

A minimum bond strength of 6 to 8 MPa is reported to be adequate for most clinical orthodontic needs [25]. These values are considered to be able to withstand masticatory and orthodontic forces. In the present work, the bond strengths of the two appliances tested onto dry enamel surface were above these limits, whereas when they were used onto water- and saliva-contaminated enamel, the minimal requirement was not achieved. In fact using disinclusion buttons bond strength values were closer than orthodontic brackets to the minimum adequate clinical values, suggesting the clinician to prefer them instead of orthodontic brackets when bonding in critical conditions onto contaminated enamel. A possible explanation could be represented by the different appliance design and/or the different mesh pad microretentions [10].

In the present investigation permanent bovine lower incisors were used. Bovine deciduous and permanent enamel has been reported to be a reliable substitute for human enamel [26, 27] in bonding studies although slightly lower [28] or significantly lower [26, 29] bonding values can be anticipated. Moreover bovine and human enamels are similar in their physical properties, composition, and bond strengths [26, 28]. Therefore, as they are readily available and inexpensive and have a close morphologic similarity to human enamel, bovine lower permanent incisors were used in the present investigation.

Moreover in the present investigation ARI scores have been recorded. Uncontaminated enamel groups reported a higher frequency of ARI score of “2.” Under contamination, conventional brackets exhibited higher frequency of ARI score of “0,” whereas disinclusion buttons showed higher frequency of ARI score of “1.” This is in agreement with previous investigations that evaluated ARI scores of orthodontic brackets bonded onto water- and saliva-contaminated enamel that all showed lower scores for moistened groups [3, 6, 9, 24]. To date in literature there are no studies that evaluated ARI scores of disinclusion buttons onto water- and saliva-moistened enamel. 

As a high number of orthodontic products is present on the market, future further studies are needed to test also other orthodontic devices bonded onto dry and contaminated enamel.

## 5. Conclusions

This study demonstrated the following.

- Noncontaminated enamel surfaces showed the highest bond strengths for both brackets and buttons. Under water and saliva contamination all groups showed significantly lower shear bond strength values.
- Under contamination orthodontic brackets groups exhibited significantly lower shear strengths than disinclusion buttons groups.
- Noncontaminated groups showed higher frequency of ARI score of “2,” both for orthodontic brackets and disinclusion buttons. For both water- and saliva- contaminated groups, orthodontic brackets reported higher frequency of ARI score of “0,” whereas disinclusion buttons displayed higher frequency of ARI score of “1.”

## Figures and Tables

**Table 1:** Bonding procedures for the different enamel surface conditions.

Appliance	Group	Bonding procedure					
Orthodontic bracket	1	Etching	Drying	Primer	—	Bonding	Light curing
	2	Etching	Drying	Primer	Water	Bonding	Light curing
	3	Etching	Drying	Primer	Saliva	Bonding	Light curing

Disinclusion button	5	Etching	Drying	Primer	—	Bonding	Light curing
	6	Etching	Drying	Primer	—	Bonding	Light curing
	7	Etching	Drying	Primer	Water	Bonding	Light curing
	8	Etching	Drying	Primer	Saliva	Bonding	Light curing

**Table 2:** Descriptive statistics (in MPa) of shear bond strengths of the 6 groups tested (each group consisted of 20 specimens).

Group	Appliance	Contamination	Mean	SD	Min	Median	Max	Tukey*
1	Orthodontic bracket	Dry environment	14.65	3.21	7.34	13.78	21.11	A
2	Orthodontic bracket	Water contamination	3.33	1.53	1.34	3.28	5.78	B
3	Orthodontic bracket	Saliva contamination	3.12	1.01	1.14	3.02	5.34	B
4	Disinclusion button	Dry environment	14.04	2.23	9.98	14.55	20.43	A
5	Disinclusion button	Water contamination	5.89	1.21	3.95	5.96	7.94	C
6	Disinclusion button	Saliva contamination	5.78	0.97	4.07	5.88	7.03	C

*Tukey grouping: means with the same letter are not significantly different.

**Table 3:** Frequency of distribution of adhesive remnant index scores (%).

Group	Appliance	Contamination	ARI = 0	ARI = 1	ARI = 2	ARI = 3
1	Orthodontic bracket	Dry environment	0 (0.0%)	1 (5.0%)	15 (75.0%)	4 (20.0%)
2	Orthodontic bracket	Water contamination	15 (75.0%)	4 (20.0%)	1 (5.0%)	0 (0.0%)
3	Orthodontic bracket	Saliva contamination	14 (70.0%)	4 (20.0%)	1 (5.0%)	1 (5.0%)
4	Disinclusion button	Dry environment	0 (0.0%)	3 (15.0%)	12 (60.0%)	5 (25.0%)
5	Disinclusion button	Water contamination	4 (20.0%)	14 (70.0%)	2 (10.0%)	0 (0.0%)
6	Disinclusion button	Saliva contamination	2 (10.0%)	16 (80.0%)	1 (5.0%)	1 (5.0%)
